# Supplementary material for: Inflammatory factors and risk of lung adenocarcinoma: a Mendelian randomization study mediated by blood metabolites
Source: Front Endocrinol (Lausanne). 2024 Aug 27;15:1446863. doi: 10.3389/fendo.2024.1446863 (PMC11384989; doi:10.3389/fendo.2024.1446863)
Supplement: Supplementary file 1 [file DataSheet1.zip › supplementary files/S2/GCST90200933/sensitivity-analysis.pdf]

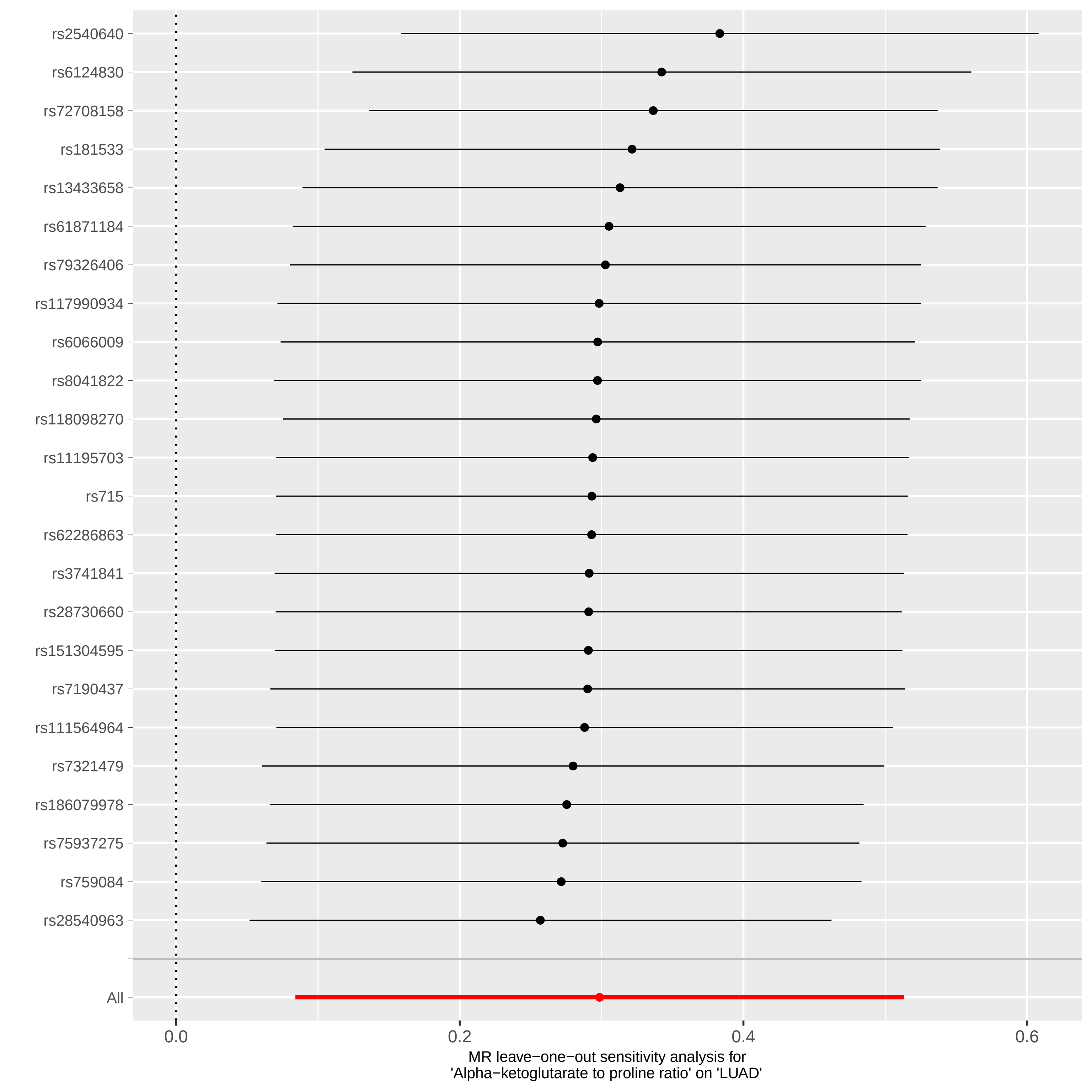
rs2540640
rs6124830
rs72708158
rs181533
rs13433658
rs61871184
rs79326406
rs117990934
rs6066009
rs8041822
rs118098270
rs11195703
rs715
rs62286863
rs3741841
rs28730660
rs151304595
rs7190437
rs111564964
rs7321479
rs186079978
rs75937275
rs759084
rs28540963
All
0.0
0.2
0.4
0.6
MR leave−one−out sensitivity analysis for
'Alpha−ketoglutarate to proline ratio' on 'LUAD'
